# ^18^F‐fluorodeoxyglucose‐positron emission tomography/computed tomography delineates involved sites in the cervical spine in Langerhans cell histiocytosis

**DOI:** 10.1002/jha2.433

**Published:** 2022-04-24

**Authors:** Shinsaku Imashuku, Hiroko Tsunemine, Chihiro Shimazaki

**Affiliations:** ^1^ Department of Pediatrics Uji‐Tokushukai Medical Center Uji Japan; ^2^ Division of Hematology Shinko Hospital Kobe Japan; ^3^ Department of Hematology Japan Community Health Care Organization Kyoto Kuramaguchi Medical Center Kyoto Japan

**Keywords:** cervical spine, FDG‐PET/CT, Langerhans cell histiocytosis

1

Langerhans cell histiocytosis (LCH), a granulomatous tumorous lesion consisting of CD1a‐positive clonal immature dendritic cells in association with various inflammatory cells, develops in tissues including bone, skin, lungs, and lymph nodes. When bone LCH exhibits osteolytic lesions, the spine is often involved, and each part of the vertebral body, vertebral arch, and vertebral spinous process can be affected. Such vertebral LCH can occur at the onset of disease and in relapse. To confirm pathological sites, a computed tomography (CT) scan is generally used to characterize lytic lesions of vertebrae, while MRI is suitable for delineating marrow and soft tissue. Additionally, to identify specific locations, ^18^F‐fluorodeoxyglucose‐positron emission tomography/CT (FDG‐PET/CT) is useful. We describe three LCH cases showing characteristic cervical spinal involvement.

**Case 1**: A 39‐year‐old woman complained of right temporal pain and was found to have osteolytic lesions of right temporal/sphenoid/petrous bones. She also had central diabetes insipidus (CDI). After the diagnosis of LCH from biopsied tissue, she was treated with chemotherapy and attained remission. Four years later, she complained of neck/shoulder pain associated with herpes zoster in the right arm. PET/CT revealed a cervical spine (C3)‐vertical body lesion (Figure 1A). With a revised chemotherapy regimen, she attained a second remission.

**FIGURE 1:**
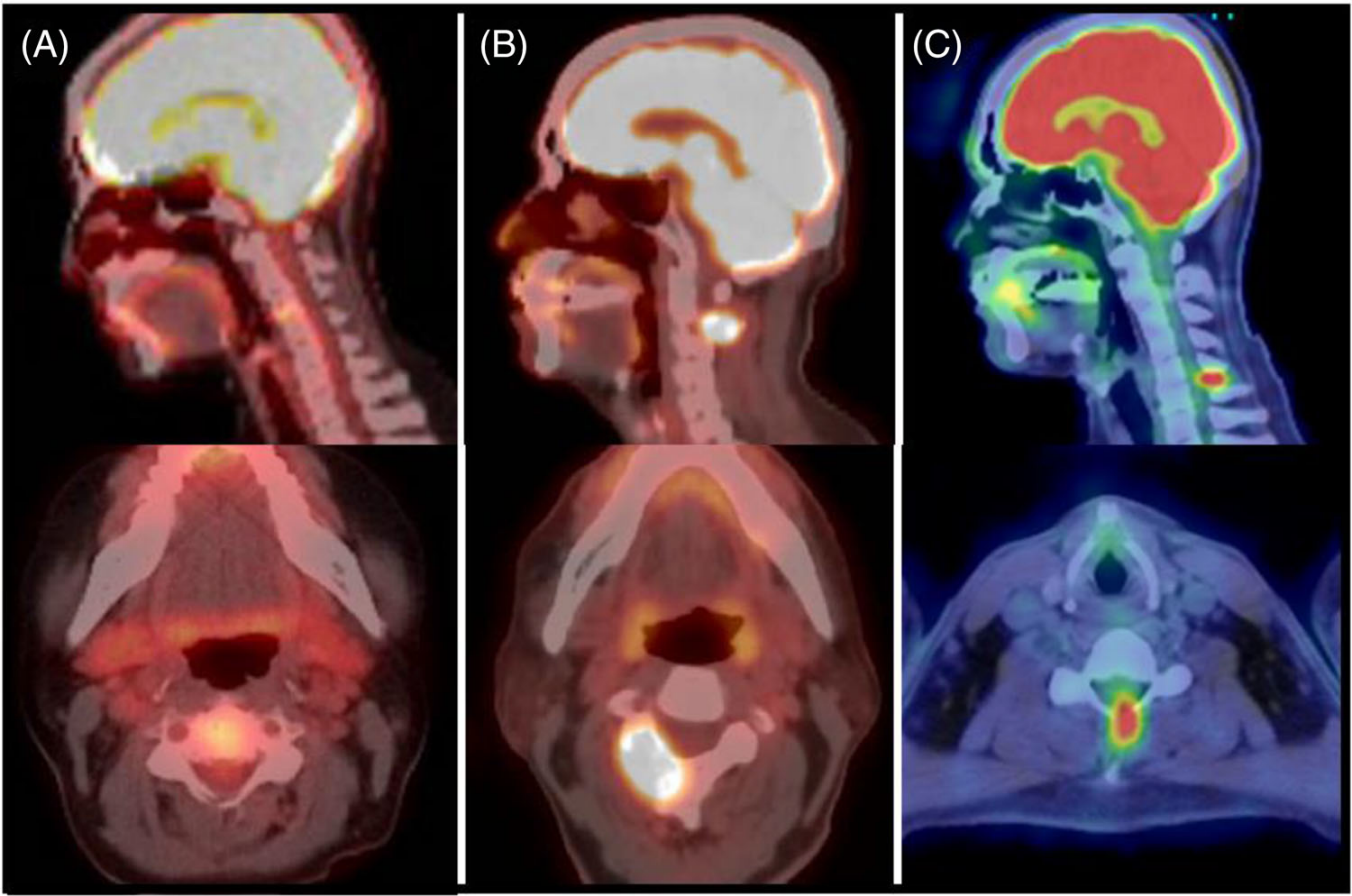
Positron emission tomography/computed tomography (PET/CT) images of three Langerhans cell histiocytosis (LCH) cases with cervical spine involvement

**Case 2**: A 70‐year‐old man, a heavy smoker for more than 40 years, complained of shortness of breath, and a chest X‐ray revealed characteristic honeycomb lungs. PET/CT showed hot lesions in the lungs, cervical spine (C3) right vertebral arch (**Figure** **1B**), and ribs. Lung lesion biopsy confirmed the diagnosis of LCH. He had no CDI and was treated with irradiation to the cervical spine and with chemotherapy.

**Case 3**: A 40‐year‐old man who had severe atopic dermatitis since the age of 20 complained of nuchal pain after a traffic accident. He was also noted to have lymphadenopathy in the bilateral axillary and inguinal areas. PET/CT revealed hot signals at the spinous process of the cervical spine (C6) (**Figure 1C**) and lymph node lesions. Biopsy of the C6 lesion confirmed LCH. He attained a remission treated with chemotherapy and zoledronic acid. Three years later, relapse at the lymph nodes was noted, but not in the C6 lesion. He was treated with chemotherapy for refractory LCH and attained a second remission.

An analysis of 76 cases of vertebral LCH by Huang et al. [1] consisted of 40 children and 36 adults, with a male to female ratio of 55:21. Lesions were predominantly in the cervical spine, followed by the thoracic and lumbosacral spines. In an analysis of 30 LCH patients with cervical spine involvement by Jiang et al. [2], 14 had atlantoaxial lesions (C1 and C2), and 16 had sub‐axial (C3–C7) lesions. Chemotherapy is recommended for the management of cervical spine lesions in cases of multifocal LCH. In our three cases, two patients had spinal involvement at onset while the other developed it at relapse. All received chemotherapy because of multifocal LCH lesions. In summary, as an imaging study of cervical spine involvement in LCH, PET/CT seems to be useful for pinpointing the involved sites of vertebrae.

## CONFLICT OF INTEREST

The authors declare no conflict of interest.
